# Increased Peripheral Blood DNA Damage and Elevated Serum Levels of Melanoma Inhibitory Activity Protein: Clues to Excess Skin Cancer Risk in Airline Pilots?

**DOI:** 10.7759/cureus.51077

**Published:** 2023-12-25

**Authors:** Piercarlo Minoretti, Miryam Liaño Riera, Andrés Santiago Sáez, Manuel Gómez Serrano, Ángel García Martín

**Affiliations:** 1 General Direction, Studio Minoretti, Oggiono, ITA; 2 Legal Medicine, Psychiatry, and Pathology, Complutense University of Madrid, Madrid, ESP; 3 Legal Medicine, Hospital Clinico San Carlos, Madrid, ESP

**Keywords:** case-control study, melanoma inhibitory activity, dna damage, skin cancer, airline pilots

## Abstract

Background and objective

The risk of malignant melanoma (MM) and other forms of skin cancer appears to be higher in airline pilots (APs), potentially due to their exposure to ionizing and ultraviolet (UV) radiation. We explored the possibility of increased peripheral blood DNA damage and elevated serum levels of the melanoma inhibitory activity (MIA) protein - a serological marker for MM known to be stimulated by UV radiation - in this professional group.

Methods

This was a case-control study involving 40 male APs, each of whom was age- and tenure-matched (≥5 years of service) with 40 male office workers (OWs). We assessed DNA damage in the two professional groups by performing comet and micronucleus (MN) assays on peripheral blood. Serum levels of MIA protein were quantified using an immunoassay.

Results

The comet tail lengths and the frequency of MN were significantly higher in APs (4.57 ± 0.79 µm and 2.05 ± 0.26 per 1000 cells, respectively) than in OWs (3.81 ± 0.60 µm and 1.76 ± 0.31 per 1000 cells, respectively, both p<0.001). Furthermore, serum MIA levels were also significantly higher in APs (7.45 ± 0.95 ng/mL) than in OWs (5.78 ± 0.54 ng/mL, p<0.001). A significant positive correlation was found between comet tail lengths in APs and their serum MIA concentrations (r=0.68, p<0.01).

Conclusions

The increased burden of DNA damage and elevated serum MIA levels in APs may offer an explanation for their higher susceptibility to MM and other types of skin cancers.

## Introduction

The initial evidence of an excess risk for specific types of cancer in commercial airline pilots (APs) was first reported in 1990 by Band et al. [1], who found an increased incidence of non-melanoma skin cancer, brain cancer, and Hodgkin’s disease. Simultaneously, Vågerö et al. [2] demonstrated that APs are among the professional groups with the highest risk of developing malignant melanoma (MM). In a comprehensive retrospective cohort study involving 458 APs, Rafnsson et al. [3] corroborated the high prevalence of MM among pilots, proposing exposure to ionizing (cosmic-type) radiation, the number of flight hours, and lifestyle factors as potential risk factors. Haldorsen et al. [4] substantiated the elevated risks for MM and non-melanoma skin cancer among Norwegian APs, while a similar study conducted in Sweden found an increased incidence of MM in commercial pilots and non-melanoma skin cancer in military pilots. In a comprehensive retrospective analysis involving 10,032 male APs over an average period of 17 years, Pukkala et al. [5] observed a significant increase in the incidence ratios of MM and non-melanoma skin cancers, particularly basal cell carcinoma. A subsequent meta-analysis by Sanlorenzo et al. [6] revealed that both APs and cabin crew members demonstrated roughly double the rate of MM compared to the general population, with mortality from MM significantly elevated among pilots. Several independent investigations have recently corroborated these findings [7-9].

The excess risk of skin cancer among APs has been traditionally attributed to their routine exposure to elevated levels of ionizing (cosmic-type) and ultraviolet (UV) radiation during flight [10,11]. However, some studies have questioned the alleged causative role of radiation exposure. Shantha et al. [12] have indicated that despite the exposure to ionizing (cosmic-type) radiation through the plane’s exterior, a 20-year career as an airline crew member only results in a relatively minor dosage. Significantly, no established connection exists between even elevated levels of this radiation and MM [12]. Additionally, Cadilhac et al. [13] found no evidence of either UVA or UVB radiation in any part of the airplane cabins tested, including the cockpit. This led them to infer that the increased incidence and associated mortality of MM among pilots and cabin crew may not directly correlate with UV radiation exposure during flights [13]. Besides the focus on cosmic radiation and UV exposure, several other potential risk factors for MM relevant to airline pilots have been suggested. These encompass skin type, the existence of atypical nevi, a family history of MM, previous episodes of severe sunburns, the use of tanning beds, and a high socioeconomic status [12].

Despite the unanswered questions surrounding the biological reasons for increased skin cancer risk, a study by Cavallo et al. [14] noted a moderate, yet not significant, increase in DNA damage among long-haul crew members compared to controls. Another study by Romano et al. [15] revealed an elevated presence of dicentric and ring chromosomes in the peripheral blood lymphocytes of flight personnel. Yong et al. [16] applied fluorescence in situ hybridization whole chromosome painting to examine the translocation frequency in 83 male airline pilots. Their findings indicated that the translocation frequency was approximately 25% higher in these pilots compared to a control group. Notably, the adjusted translocation frequency showcased a positive correlation with the cumulative number of flight years among the pilots [16]. Adding to the discourse, several authors proposed a hypothesis that the occupational hazards associated with aircrew work might potentially compromise genomic integrity [17,18]. Starting from these premises, we designed the present investigation to further elucidate the extent of DNA damage experienced by APs. By employing a case-control design, we examined 40 male APs, each of whom was age and tenure-matched (≥5 years of service) with 40 male office workers (OWs). The burden of DNA damage in the two professional groups was assessed by performing comet and micronucleus (MN) assays on peripheral blood. Finally, serum levels of the melanoma inhibitory activity (MIA) protein - a serological marker for MM [19] known to be stimulated by UV radiation [20] - were also determined.

## Materials and methods

Participants

In this case-control study, a convenience sample of 80 male participants was selected, spanning two distinct professional fields - APs and OWs - with each group comprising 40 individuals. These subjects were voluntarily recruited during routine occupational health assessments conducted at outpatient clinics (Studio Minoretti, Oggiono, Italy), with invitations to participate disseminated by an occupational health physician. All participants were of Caucasian descent. To mitigate potential confounding variables, each OW was matched to an AP in terms of age and tenure, requiring at least five years of service. The exclusion of women from the study was due to the limited female representation within the APs population. Individuals with a history of inflammatory, autoimmune, or infectious diseases were excluded, as were those with malignancies or who had undergone drug therapy within the previous 90 days. Additionally, none of the participants were consuming any dietary supplements, and they all appeared to be in good physical health. Before their engagement in the study, every participant provided written informed consent. To mitigate the confounding impact of seasonal variations, blood sampling for this study was carried out during the winter months, specifically between November and January. The research adhered to the ethical standards set forth by the Declaration of Helsinki, with the protocol receiving approval from the local ethics committee (Studio Minoretti; reference number: 2021/08).

Assessment of DNA damage

A sample of blood (40 µL) collected from each participant was utilized for the comet assay, which was conducted under alkaline conditions as previously described [21]. A trypan-blue exclusion assay was used to determine cell viability, which ranged from 95% to 97%. This ensured that only viable cells were used for the assay. For each subject, duplicate slides were prepared and examined under a 400× magnified Olympus BX 51 fluorescence microscope (Olympus, Tokyo, Japan), equipped with an excitation filter of 515-560 nm, and a barrier filter of 590 nm. Slides were coded and arranged randomly to prevent any bias. Scoring was carried out by a single pathologist to eliminate the potential for inter-scorer variability. For each subject, 100 cells were examined (50 cells from each slide). Cells exhibiting no damage had intact nuclei without tails, while damaged cells displayed a comet-like appearance. The comet tail length, representing the extent of DNA damage, was measured using an ocular micrometer. The length of the DNA that had migrated in the comet tail was calculated as the maximum total length minus the head diameter. In the MN assay, we established lymphocyte cultures for each participant. This involved adding 0.5 mL of whole blood to 5 mL of Gibco™ PB-MAX™ Karyotyping Medium (Thermo Fisher Scientific, Waltham, MA), supplemented with 2% phytohemagglutinin M. After 44 hours of culture, we introduced cytochalasin B (6 mg/mL; Sigma, St. Louis, MO) to block cytokinesis, which allowed identifying the dividing lymphocytes. The culture was subsequently maintained at a temperature of 37 °C for 72 hours. Subsequently, cells were exposed to a hypotonic treatment utilizing 0.075 M KCl for five minutes at room temperature, followed by fixation in a methanol:acetic acid solution (v/v, 3:1). In the final step, the cells were carefully spread onto slides and stained with a 5% Giemsa solution in a phosphate buffer (pH 6.8) for five minutes. This process enabled us to identify cells that had completed the first mitosis, recognized as binucleated cells, and selectively screened for the presence of MN. For each case, approximately 1000 binucleated cells were scrutinized for MN.

Quantification of serum MIA levels

Venous blood samples underwent centrifugation at 2000 g for 15 min. The resulting serum samples were stored at −80 °C, ensuring they were only thawed out immediately before analysis. The measurement of serum MIA levels was performed using a commercially available ELISA kit according to the manufacturer’s protocol (Roche Diagnostic GmbH, Mannheim, Germany). The inter- and intra-assay coefficients of variation were less than 8% and 6%, respectively. The samples were arranged in a randomized sequence within various batches, with laboratory staff blinded to the participants’ professional status. Every sample from individual subjects was analyzed in duplicate within the same assay.

Statistical analysis

The Kolmogorov-Smirnov test was employed to assess the normality of continuous data. The results indicated that all variables adhered to a normal distribution, thus justifying the exclusive use of parametric statistical methods. Continuous data are expressed as the mean ± standard deviation (SD), whereas categorical data are presented as counts and percentages. A comparative analysis of variables between APs and OWs was conducted using the Student’s t-test for continuous data, and the chi-squared test for categorical variables. We initially performed correlation analyses, utilizing Pearson’s correlation coefficient, and then proceeded with multivariable linear regression to ascertain the independent associations between measures of DNA damage and serum MIA levels in APs. The following potential confounding factors were entered into the multivariable linear regression model: age, length of service, body mass index, total cholesterol, smoking status, and fasting plasma glucose. All analyses were performed using the SPSS Statistics software, version 20.0 (IBM, Armonk, NY). Statistical significance was determined by a two-tailed p-value <0.05.

## Results

The study included 40 APs and 40 OWs; their general characteristics are outlined in Table 1.

**Table 1:** General characteristics of the study participants Data are expressed as mean ± standard deviation unless otherwise indicated NS: not significant

Variable	Airline pilots (n=40)	Office workers (n=40)	P-value
Men, n (%)	40 (100%)	40 (100%)	NS
Age, years	39.2 ± 3.3	38.9 ± 3.4	NS
Length of service, years	9.8 ± 3.8	10.2 ± 4.1	NS
Current smoking, n (%)	7 (17.5%)	8 (20.0%)	NS
Body mass index, kg/m^2^	24.3 ± 2.4	24.6 ± 2.7	NS
Total cholesterol, mg/dL	210 ± 11	214 ± 10	NS
Fasting plasma glucose, mg/dL	90 ± 9	92 ± 11	NS

The groups were statistically similar in terms of age, length of service, current smoking habits, body mass index, total cholesterol, and fasting plasma glucose levels.

**Figure 1:**
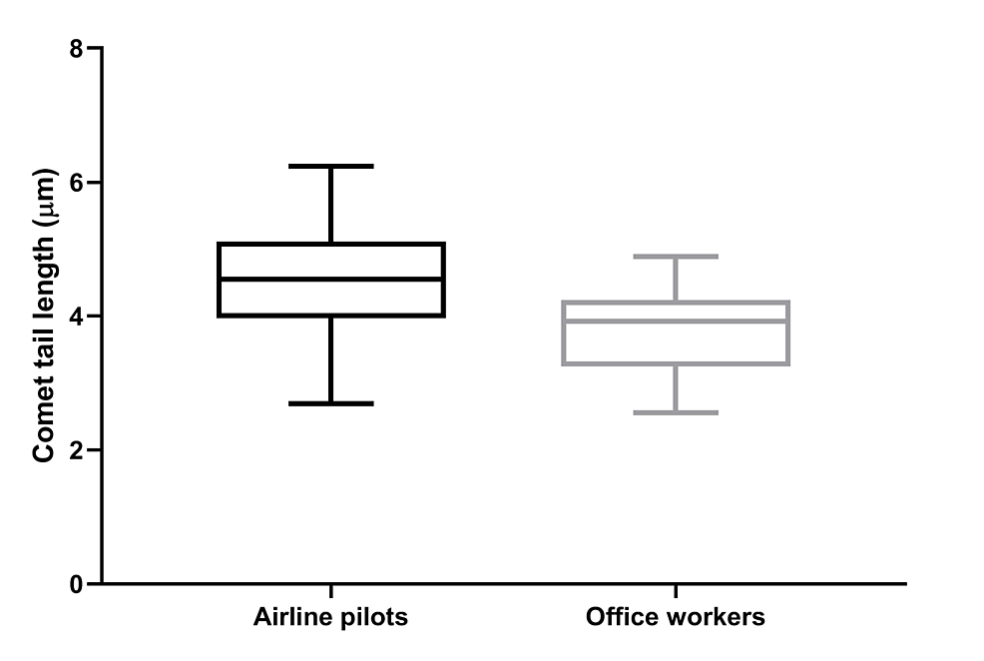
Box and whisker plots of comet tail lengths in airline pilots and office workers

We observed a consistently higher degree of DNA damage in the peripheral blood of APs than in OWs, as assessed by the comet and MN assays. In particular, APs exhibited significantly greater comet tail lengths (4.57 ± 0.79 µm) compared to the OWs (3.81 ± 0.60 µm, p<0.001) (Figure 1).

**Figure 2:**
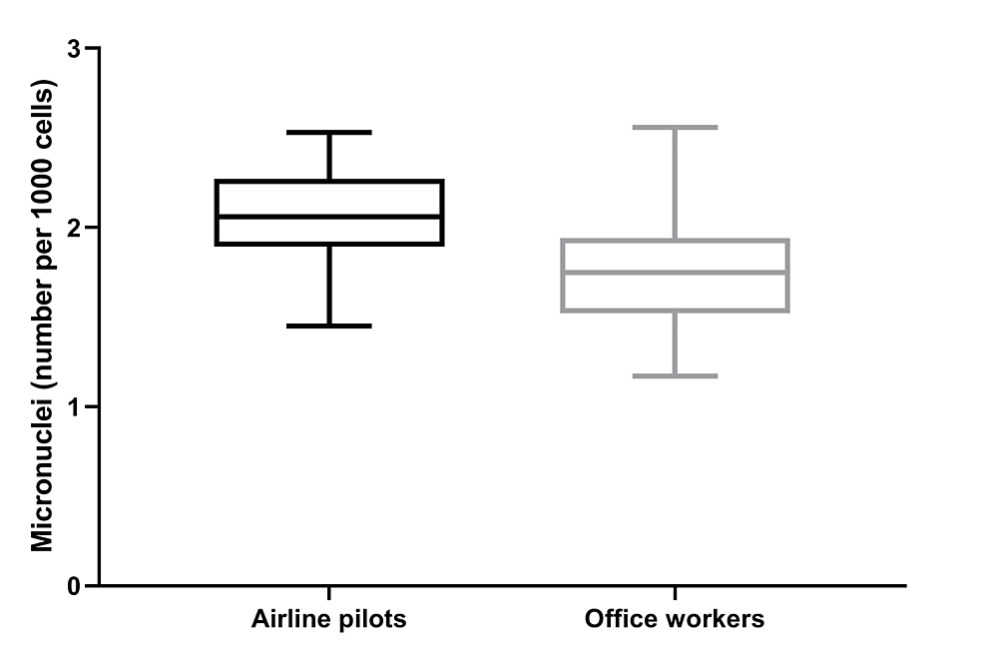
Box and whisker plots of micronuclei frequency in airline pilots and office workers

Similarly, the MN assay revealed a higher MN frequency in APs (2.05 ± 0.26 per 1000 cells) versus OWs (1.76 ± 0.31 per 1000 cells, p<0.001) (Figure 2).

**Figure 3:**
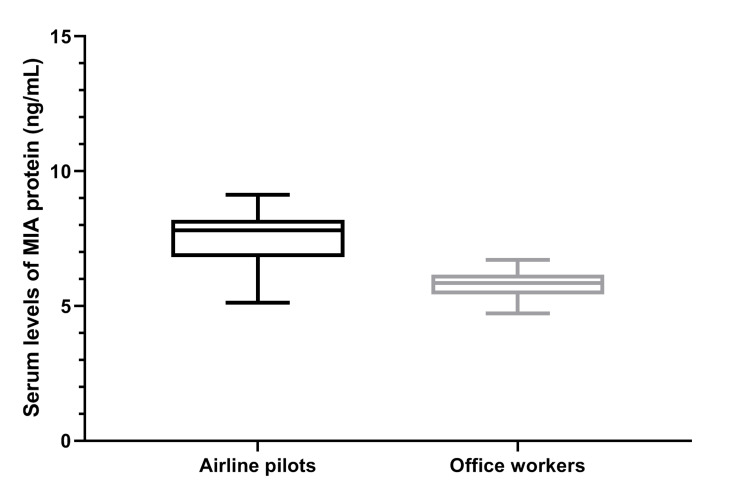
Box and whisker plots of serum melanoma inhibitory activity concentrations in airline pilots and office workers

Additionally, APs had significantly elevated serum MIA levels (7.45 ± 0.95 ng/mL) compared with OWs (5.78 ± 0.54 ng/mL, p<0.001 (Figure 3).

**Figure 4:**
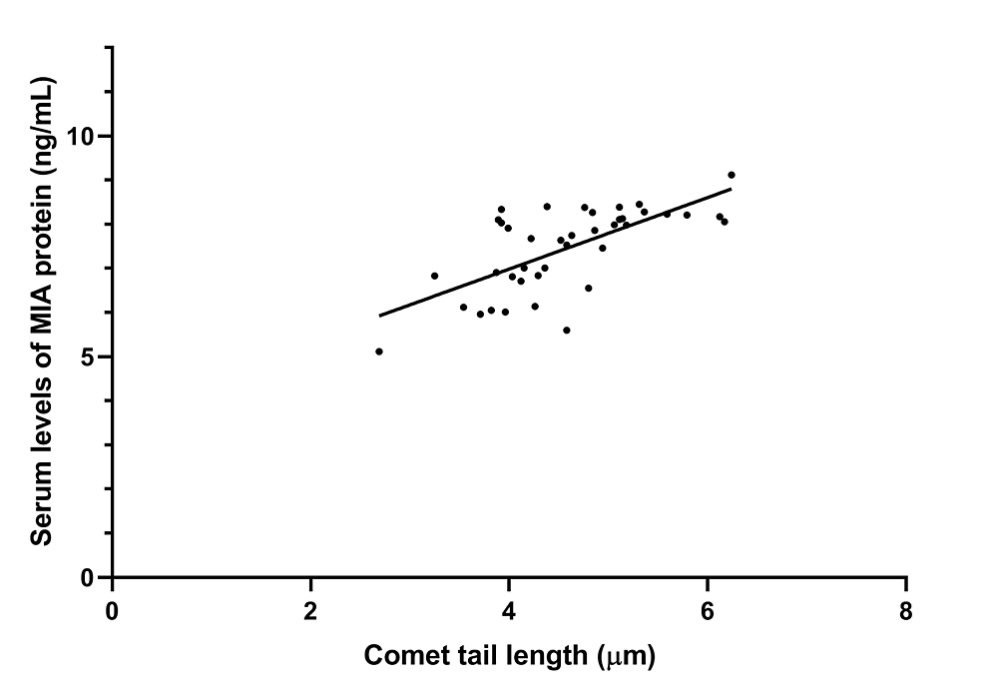
Scattergram and regression line depicting the positive relationship between the comet tail lengths measured in APs and their serum MIA concentrations APs: airline pilots; MIA: melanoma inhibitory activity

We detected a significant positive correlation between the comet tail lengths of APs and their serum MIA concentrations (r=0.68, p<0.01 (Figure 4).

However, no other significant correlations were observed. Of note, the link between APs’ comet tail lengths and their serum MIA concentrations was found to be independent of potential confounders, as revealed by multivariable linear regression analysis (beta=0.62, t=5.63, p<0.01).

## Discussion

Based on the data derived from the comet and MN assays, our study indicates a significant increase in peripheral blood DNA damage in APs compared to OWs. Moreover, we observed elevated serum MIA levels in APs, with a significant, independent association between the circulating concentrations of this protein and comet tail lengths. MIA is a protein primarily hyperexpressed in MM [19], although it is also moderately expressed in a subset of normal melanocytes [20]. By engaging with key components of the extracellular matrix, MIA has been shown to efficiently hinder the adhesion of suspended malignant cells to surfaces coated with fibronectin and laminin. This interference remarkably enhances the cells’ capacity for dissemination, thereby potentially augmenting tumor invasiveness [21]. Consequently, an increased expression of MIA in melanoma cells could suggest a more aggressive biological behavior, which could escalate the malignancy’s invasive potential [22-24].

However, MIA levels serve not only as a tumor marker in MM but also exhibit an elevation in healthy individuals following exposure to UV radiation, one of the most formidable skin carcinogens. This correlation has been substantiated in a study by Datz et al. [19] who found that UV phototherapy administered to patients with non-malignant dermatological conditions precipitated an increase in serum MIA concentrations. This elevation in MIA levels was construed as serological proof highlighting the overall tumor-promoting attributes of UV radiation [19]. This is particularly significant as UV exposure is strongly associated with the development of skin malignancies [25]. The observed elevation in serum MIA levels among our APs, compared to those engaged in an indoor profession such as office work, could potentially be attributed to their increased exposure to both ionizing (cosmic-type) and UV radiation. Indeed, these types of radiation are known to stimulate a transient activation of melanocytes [26].

Regrettably, we were unable to determine if the increase in serum MIA levels in APs represents a temporary or a persistent phenomenon over time. Addressing this question could yield intriguing insights pertinent to clinical implications. Concurrent with the increased expression of MIA, our study corroborates the existing evidence that APs may bear a higher burden of DNA damage in their peripheral blood [14-16]. This cluster of findings collectively implies that this occupational group exhibits biological indicators of radiation exposure, which are detectable at both the DNA and protein (specifically MIA) levels. While we must emphasize that we cannot definitively ascertain if this exposure is professional (in-flight) in nature, it is also plausible that the affluent status of APs, which frequently permits recreational sun exposure, may be a contributing factor to these observed signatures. Whether the exposure source is professional or recreational, our findings suggest that a high burden of DNA damage and increased MIA levels could potentially serve as an underlying mechanism for the higher skin cancer incidence rates observed in APs.

The primary strength of our study lies in its comprehensive exploration of different molecular outcomes, with a focus on uncovering the mechanisms that could potentially link an airline pilot's profession to skin cancer. However, our research has several limitations. The limited sample size represents an element of uncertainty, preventing us from drawing definitive conclusions. Furthermore, the voluntary involvement of APs and OWs might limit the generalizability of our findings due to the self-selection bias. While the sex distribution of our sample reflects that of the broader airline pilot demographic [27], the lack of female participants hinders the relevance of our results to women. Incorporating a quantifiable evaluation of professional radiation exposure could considerably enhance the reliability and validity of our conclusions. Finally, the observed changes in biomarkers require further longitudinal and prospective exploration to gain a more comprehensive understanding.

## Conclusions

Our study, albeit limited by a small sample size, suggests a potential link between the profession of airline piloting and molecular changes that could lead to skin cancer, setting them apart from OWs. To validate these initial findings, we recommend conducting more comprehensive studies involving larger workforce demographics. Improving exposure assessment methods, particularly through direct in-flight radiation exposure measurements, could significantly enhance the accuracy and validity of our results. Future research could also focus on the longitudinal monitoring of serum MIA concentrations in pilots. For example, it would be interesting to measure these levels not only during their active flying careers but also post-retirement. This would provide valuable insights into the variations of serum MIA over time and its persistence beyond the period of occupational exposure.

In addition to longitudinal assessment, a comparative analysis of serum MIA levels between active and retired pilots could further elucidate the occupational impacts. This comparison could help determine if the cessation of flying duties correlates with a decrease in serum MIA levels, suggesting a potential link between active flying and elevated levels of this marker. Another crucial aspect for future research is to establish whether a correlation exists between elevated serum MIA levels and the development of skin malignancies, specifically melanoma, in pilots. By monitoring the incidence of skin cancer in this population and correlating it with their serum MIA levels, we could assess if high serum MIA is a predictive biomarker. If a significant correlation is found, serum MIA could serve as a marker to identify pilots at a higher risk of developing skin malignancies, potentially leading to earlier interventions and customized skin protection measures.
